# Comparative Study of Approaches for Detecting Crime Hotspots with Considering Concentration and Shape Characteristics

**DOI:** 10.3390/ijerph192114350

**Published:** 2022-11-02

**Authors:** Zhanjun He, Rongqi Lai, Zhipeng Wang, Huimin Liu, Min Deng

**Affiliations:** 1 School of Computer Science, China University of Geosciences, Wuhan 430074, China; 2 Artificial Intelligence School, Wuchang University of Technology, Wuhan 430223, China; 3 State Key Laboratory of Geo-Information Engineering, Xi’an 710054, China; 4 Department of Geographic Information, Central South University, Changsha 410083, China

**Keywords:** criminal geography, crime pattern, hotspots detection

## Abstract

Hotspot detection is an important exploratory technique to identify areas with high concentrations of crime and help deploy crime-reduction resources. Although a variety of methods have been developed to detect crime hotspots, few studies have systematically evaluated the performance of various methods, especially in terms of the ability to detect complex-shaped crime hotspots. Therefore, in this study, a comparative study of hotspot detection approaches while simultaneously considering the concentration and shape characteristics was conducted. Firstly, we established a framework for quantitatively evaluating the performance of hotspot detection for cases with or without the ”ground truth”. Secondly, accounting for the concentration and shape characteristics of the hotspot, we additionally defined two evaluation indicators, which can be used as a supplement to existing evaluation indicators. Finally, four classical hotspot-detection methods were quantitatively compared on the synthetic and real crime data. Results show that the proposed evaluation framework and indicators can describe the size, concentration and shape characteristics of the detected hotspots, thus supporting the quantitative comparison of different methods. From the selected methods, the AMOEBA (A Multidirectional Optimal Ecotope-Based Algorithm) method was more accurate in describing the concentration and shape characteristics and was powerful in discovering complex hotspots.

## 1. Introduction

In the research of environmental criminology, crime hotspot has always been a concept that has received wide attention [1]. Crime hotspots are even regarded as the most basic and important indicator that summarize the inhomogeneity of crimes [2]. Crime hotspot detection also plays an important role in crime prediction and crime prevention [3]. On the one hand, it helps in identifying high-risk locations or regions that require further attention or even intervention [4,5]. On the other hand, crime hotspots can help reveal the influencing factors of crime concentration [6,7]. This can bring some enlightenment to the deployment of resources and formulation of policies. Currently, there is no clear definition for crime hotspots, and the term ”hotspot” has several meanings. Generally, crime hotspots can be understood in two ways. Firstly, crime hotspots refer to areas where crimes are concentrated [8]. To detect the crime hotspots, methods such as hotspot mapping (e.g., kernel density estimation) and clustering (e.g., K-means clustering, hierarchical clustering) can be applied [9,10,11,12]. Secondly, crime hotspots require not only the clustering tendency of crimes, but also the significant concentration. In this way, hotspots refer to high-value clusters of crime cases, and the significance of clusters can be measured by some spatial statistics. These types of methods include the spatial scan statistic, local Moran’s *I* and Getis–Ord G* [13,14,15]. The calculation of statistics, such as local Moran’s *I* and Getis–Ord G*, are based on areal data with a specific attribute, such as crime counts, crime rates and even kernel density values [16,17,18].

Despite the fact that numerous hotspot-detection methods exist to support crime-concentration analysis, there are still some challenges to be solved. One major challenge is determining which approaches are suitable for particular problems and application contexts, that is, to answer questions such as “what are the differences between different methods in detecting hotspots” and “how to choose a suitable hotspot-detection method to support the police practice”. To answer the question, it is necessary to compare the performance of different hotspot detection methods comprehensively. However, there are few studies on comparative analysis of crime hotspot detection due to the following difficulties. Firstly, there is no clear definition of crime hotspots, and the term “hotspot” has multiple meanings. Meanwhile, hotspot detection is essentially an unsupervised method, which lacks true or labelled clusters to judge the detected results. As a result, there is no evaluation benchmark (or “ground truth”) to compare the performance of different methods. Some studies only made a visual comparison of the hotspots detected by different methods [19,20,21]. Obviously, a visual comparison is often subjective and the accuracy of detected hotspots is difficult to measure. Other studies tried to compare the performance of hotspot detection methods by jointly considering the number of crime incidences and spatial areas captured by the detected hotspots [22]. For example, Bowers et al. [23] used indicators such as the hit rate, occupied area and search efficiency (the number of cases per unit area) to evaluate the utility of different hotspots; Chainey et al. [24] proposed using the prediction accuracy index (*PAI*) to measure the prediction accuracy of identified hotspots. Specifically, the detected hotpots in historical data are also regarded as predicted hotspots, while the actual crime incidents located in the hotpots in the next timestamp are used as the predicted crime incidents. *PAI* takes into account the area ratio of hotspots while measuring the capture ability of hotspots and is widely used in hotspot evaluation [25,26,27,28]. Further, Johnson et al. [29] proposed the use of an accuracy concentration curve. An accuracy concentration curve is generated by plotting the percentage of crimes that have been accurately predicted against the incremental risk ordered percentage of the study area. The main problem that hinders the evaluation research of crime hotspot detection lies in the uncertainty surrounding the definition of “hotspot”. As described above, the hotpots refer to spatial regions where the crime cases are clustered. However, the extent to which the crime is clustered can be considered as a hotspot and the shape characteristics of crime hotspots is seldom discussed. Obviously, the shape of clusters is not limited to the regular form (e.g., squares or circles). In addition, the incidence of crime may be clustered in a complex shape. To evaluate the utility of crime hotspot detection method comprehensively, the accuracy and shape characteristics of detected hotpots should be considered.

To solve the aforementioned problems, this study conducted a comparative study of approaches to detecting crime hotspots by considering the concentration and shape characteristics. This article is structured as follows. In Section 2, the existing research is reviewed, while several hotspot detection methods that were used for comparison are briefly introduced. In addition, the specific indicators used to evaluate hotspots are described in detail. In Section 3, a hotspot detection and evaluation analysis is conducted on simulated data and real crime data, respectively. Finally, the characteristics of different methods are discussed and summarized.

## 2. Materials and Methods

### 2.1. Hotspot Detection Methods

As mentioned above, crime hotspots can be understood in two ways. Correspondingly, methods for hotspot detection can be classified into two categories, i.e., the clustering-based methods and statistics-based methods. For the statistics-based methods, the detected hotspots are statistically significant and can describe the distribution characteristics of criminal incidents in an objective way. Therefore, we discriminated the two kinds of methods in this study and only focused on the statistics-based methods. According to the adopted statistics, the statistics-based methods can be further divided into two categories, scan-based and autocorrelation-based approaches [30]. Scan-based approaches for hotspot detection are implemented based on a geographical “window”, which is used to identify areas with elevated rates of crime incidences. Popular work includes Openshaw et al. [31], Besag and Newell [32], Fotheringham and Zhan [33], and Kulldorff [13]. The autocorrelation-based approach, just as its name indicates, is mainly based on the local spatial autocorrelation statistic, for example, the local Moran’s *I* and Getis–Ord G*. To implement the local spatial autocorrelation statistic, the spatial neighborhood should be constructed first. Usually, the spatial neighborhood is constructed in a regular way (e.g., the rook criterion). However, regular spatial neighbourhood in the traditional local spatial autocorrelation statistic does not guarantee the optimal spatial partition, as some studies also try to find the hotspot by a multi-directional optimum ecotope-based algorithm, i.e., the AMOEBA method [34].

To compare the performance of different hotspot detection methods, several classical statistics-based methods were selected, including a scan-based approach (i.e., the spatial scan statistic) and three autocorrelation-based approaches, i.e., the local Moran’s *I*, Getis–Ord G* and the AMOEBA methods. The selected methods are described briefly in below.

(1) Spatial scan statistics

Spatial scan statistics is a classical hotspot detection method, which detects the spatial clustering using a series of scanning circles [13]. Currently, the spatial scanning statistical method is widely used in recognition of spatial patterns of crimes [35,36,37,38,39]. Generally, spatial scanning statistical methods comprise three steps: determining the scanning window, calculating statistics and testing the significance. The spatial scan statistic imposes a circular window on the map. The window is in turn centered on each case point or the center point of an area unit positioned throughout the study region. For each point, the radius of the window varies continuously in size from zero to upper limit. In this way, the circular window is flexible both in location and size. Then, based on the scan window, the spatial statistics are defined and calculated. Finally, the significance of the alternative hypothesis is tested (H1: at least one crime rate within the scanning circle is significantly higher than outside the scanning circle). The test method calculates the likelihood ratio between the actual value and the expected value of the case inside and outside of each scan circle and determines the scan radius that maximizes the likelihood ratio of each scan circle, thereby obtaining potential clusters. Common spatial scanning statistical models include the Bernoulli, Poisson [13], ordinal [40], exponential [41], normal [42] and multinomial models [43]. For example, the likelihood ratio (*LR*) of the Poisson distribution is calculated as follows:

(1) $$LR={\left(\frac{c}{\mu }\right)}^{c}{\left(\frac{C-c}{C-\mu }\right)}^{C-c}$$

where *C* and *c* are the actual number of cases in the area and circle, respectively; *μ* is the expected number of cases in the scanning circle. The ratio *LR* is used as the test statistic and its significance is tested by Monte Carlo simulation. Monte Carlo testing is a numerical method based on random trials and statistical calculations and is widely used to evaluate the significance of selected spatial statistics [44]. The idea of the Monte Carlo testing is randomly rearranging the cases in the study region N times (e.g., N = 999) to form N random simulated spatial distributions. Then, the likelihood ratio for each scanning circle on the simulated distribution can be calculated. By ranking the likelihood ratios in ascending or descending order, the statistical significance of the spatial statistics can be obtained.

(2) Getis–Ord G* Statistic

The Getis–Ord G* Statistic, developed by Getis and Ord, aims to identify the spatial patterns of “hotspots” or “cold spots” [15]. Research on the application of this method to detect crime hotspots includes Frazier [16], Malleson [45], Ceccato [46], Malleson [47] and Haleem [48]. The idea of the method is to determine the clusters of high values by comparing the sum of attribute values in a spatial local region with the sum of the attribute values in the whole study area. Given an area subdivided into n regions, *i* = 1, 2, …, *n*, where each region *i* has associated with the attribute value *x*. The $G_{i}^{*}\left(d\right)$ statistic can be written as

(2) $$G_{i}^{*}\left(d\right)=\frac{\sum_{j=1}^{n}w_{ij}\left(d\right)x_{j}}{\sum_{j=1}^{n}x_{j}}$$

where *d* is the distance threshold for defining the spatial proximity and $\left\{w_{ij}\right\}$ is the weight matrix describing the spatial relation between regions *i* and *j* (i.e., within distance *d*). If the $G_{i}^{*}\left(d\right)$ statistic is different from the expected value, it is reasonable to interpret it as a significant spatial cluster. Usually, the significance of the cluster is tested by the *Z*-value, which is formulated as follows:

(3) $$Z_{G_{i}^{*}\left(d\right)}=\frac{G_{i}^{*}\left(d\right)-E\left(G_{i}^{*}\left(d\right)\right)}{\sqrt{var\left(G_{i}^{*}\left(d\right)\right)}}$$

If the significance test is passed, it indicates that the *x* value of the area around the region *i* forms a spatial cluster.

(3) The local Moran’s *I*

Local Moran’s *I* is used as a local indicator of spatial association [14] and is also widely used to determine where crimes are spatially clustered [17,49,50,51,52]. The idea of Moran’s *I* is comparing the difference of an attribute value in a spatial neighborhood with that in the study area. The statistic can be written as

(4) $$I_{i}=\frac{x_{i}-\bar{x}}{S^{2}}\sum_{j}^{n}w_{ij}\left(x_{j}-\bar{x}\right)$$

where $\bar{x}$ and $S^{2}$ are the mean and variance of $x_{i}$, respectively. The sign of $I_{i}$ depends on $x_{i}-\bar{x}$ and $\sum_{j}^{n}w_{ij}\left(x_{j}-\bar{x}\right)$ simultaneously. The former reflects the similarity between the attribute value of the *i*-th region and the average value of the entire study area, while the latter can reflect the similarity between the surrounding area of the *i*-th region and the entire area. Just as with Gi*, the significance of $I_{i}$ is tested by *Z*-value, which is formulated as:

(5) $$\frac{I_{i}-E\left(I_{i}\right)}{\sqrt{var\left(I_{i}\right)}}∼N\left(0,var\left(I_{i}\right)\right)$$

If the significance test is passed, and $I_{i}$ and $x_{i}-\bar{x}$ are both greater than zero, it indicates that there is a high-value cluster in the *i*-th region.

(4) A Multidirectional Optimal Ecotope-Based Algorithm

The AMOEBA is a multidirectional optimal ecotope-based algorithm that aims to find the optimal clusters on the basis of the Getis–Ord G*. For the *i*-th unit, the algorithm generates a different combination of spatial neighborhoods (termed as the ecotope) and finds the combination that maximizes $G_{i}^{*}$ [34]. The general steps of the method can be illustrated by the local statistic $G_{i}^{*}$. First, calculate the $G_{i}^{*}$ statistics at the position i and mark it as $G_{i}^{*}\left(0\right)$. At this time, the ecotope comprises the *i*-th unit. Next, combine the *i*-th unit and its first-order neighbors, and calculate $G_{i}^{*}\left(0\right)$ for each possible combination, denoted as $G_{i}^{*}\left(1\right)$. The combined area with the largest $G_{i}^{*}\left(1\right)$ value becomes a new ecotope and the spatial units excluded in this combination do not participate in further calculations. For the new ecotope, repeat the process above to identify new ecoregion members. The final ecotope can be obtained when increasing any continuous unit would not increase the absolute value of $G_{i}^{*}$. The significance of the final ecotope can be checked by Monte Carlo testing. In previous research, the AMOEBA approach was also applied to detect and visualize crime clusters [53].

### 2.2. Framework for Evaluating Performance of Spatial Hotspot Detection

Due to the lack of ground truth about crime hotspots, it is necessary to design appropriate evaluation criteria to describe the utility of the detected hotspots. To evaluate the performance of hotspot detection methods comprehensively, at least three aspects should be considered. Firstly, the hotspot area should capture as many crime cases as possible. This is the most basic evaluation criterion for performance of hotspot detection. However, the number of cases does not guarantee the “hot” regions. Assuming an extreme case, a hotspot covering the entire study area would capture all crime cases, but such a hotspot is limited in explaining the uneven spatial distribution pattern of crime incidents. Therefore, it is necessary to consider the number of crimes captured by hotspots and the area occupied by hotspots. Inspired by the existing hotspot prediction evaluation indicators, the following indicators are used to evaluate the performance of hotspot detection.

(6) $$HitRate=\frac{n}{N}$$

(7) $$AreaRatio=\frac{a}{A}$$

(8) $$PAI=\frac{\left(\frac{n}{N}\right)*100}{\left(\frac{a}{A}\right)*100}=\frac{HitRate}{AreaRatio}=\frac{n/a}{N/A}$$

where *A* denotes the area of the study region; a refers to the area of detected hotspots; *N* is total number of cases in the study area; and *n* is the number of cases in the hotspot regions. Obviously, the *HitRate* measures the proportion of crime cases in the hotspot area to the total cases in the study area, and the *AreaRatio* refers to the percentage of the area of the hotspot area to the area of the study area.

The *PAI* is calculated by dividing the *HitRate* by the *AreaRatio*. The purpose of this index is to consider both the hit rate and the proportion of hotspot area when measuring performance of hotspot detection. In fact, *PAI* can also be interpreted as the ratio of the case density in the hotspot area to that in the entire study area (see Equation (8)). For the same research area and case points, the denominator of the *PAI* is a constant. Therefore, the *PAI* can be understood as the standardization of the crime case density in hotspot.

The second issue in evaluating performance of hotspot detection is the high concentration of crime cases. Although the hit rate and *PAI* can reflect the crime concentration to some extent, this still suffers some limitations. Firstly, the hit rate and *PAI* may be contradicted with each other. For example, abandoning some areas with relatively low crime density will result in a higher *PAI* and reduce the number of captured cases simultaneously. Secondly, all indicators are unable to reveal the true meaning of hotspots. A hotspot is a relative concept. It could be viewed as significant “clusters” in the foreground, which is compared with the non-clustered distribution in the background. Therefore, an intuitive way to evaluate hotspots is based on the difference between hotspots (i.e., the foreground) and non-hotspot regions (i.e., the background). Based on these concerns, a new index called the density contrast ratio (*DCR*) was defined as a supplement to existing hotspot evaluation indicators, which can be formulated as follows:

(9) $$DCR=\frac{n/a}{\left(N-n\right)/\left(A-a\right)}$$

The *DCR* refers to the ratio of the case density in the hotspot to that outside the hotspot region. Density contrast ratio is greater when the density of the hotspot area is higher and the density outside the hotspot area is lower. That guarantees that the detected hotspots are “hotter” than other regions.

The third issue to be considered is the ability to describe the complex shape of hotspots. All indicators mentioned above only focus on the geographic location and concentration of crime incidents, neglecting the geometric properties of the hotspot (e.g., the shape characteristic). If the detected hotspot has a complex shape, on the one hand, it means that the detection method has an excellent ability to capture complex shapes; on the other hand, such a hotspot may be detrimental to the deployment of police resources. In previous research, the indicator of area-to-perimeter ratios was commonly used to measure the geometric complexity of hotspots (i.e., the compactness) [23]. However, the area-to-perimeter ratio has some drawbacks. Firstly, the ratio has a length unit and its value is related to the scale of the study area, making the cross-regional comparison unreasonable. Secondly, the theoretical value of the perimeter-to-area ratio can be infinite, making it difficult to interpret values of the ratio [54]. To overcome the shortcomings of the area-to-perimeter ratio, we may refer to the square pixel index (*SQP*) in landscape ecology and formulate a standardized shape index to describe the shape characteristics of hotspots [55]. The *SQP* considers the perimeter area relationship for raster data structures and normalizes the ratio of perimeter and area to a value between 0 and 1 [56]. Change the *SQP* reference from a square to a circle and slightly modify its form to obtain a standardized shape index (*SSI*), which can be formulated as follows:

(10) $$SSI=1-\frac{2\sqrt{\pi a}}{P}$$

where *P* represents the perimeter of the hotspot area and $\frac{P}{2\sqrt{\pi a}}$ refers to the landscape shape index (*LSI*) commonly used in landscape ecology [57]. The simpler the patch shape, the smaller the *LSI* index is. When the shape of the patch is simple enough (i.e., a circle), the *LSI* index is equal to 1. Obviously, *SSI* is a normalized form of *LSI*, with values ranging from 0 to 1. The more complex the shape of the hotspot, the larger the index is, which theoretically approaches one infinitely.

The index is essentially a comparison of the perimeter-area ratio of patch to that of circles. Therefore, when the shape of a hotspot cluster is circular, the *SSI* is the minimum value of 0. There are often multiple hotspots in the research field. Therefore, we used the sum of the perimeters of all hotspots and the sum of the areas of all hotspots to calculate the overall shape index.

**Figure 1:**
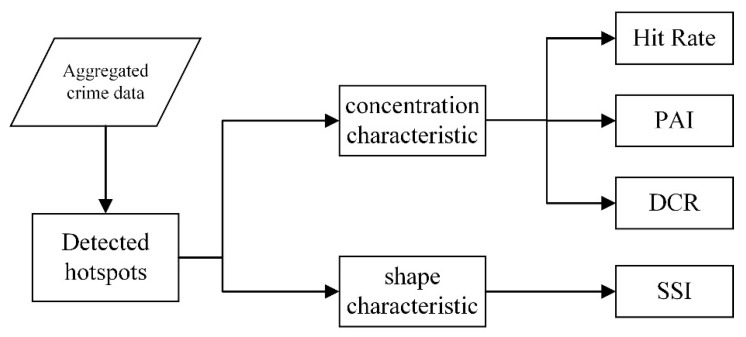
Crime hotspot evaluation framework.

In summary, we propose a system to evaluate the effectiveness of different hotspot detection methods, as shown in Figure 1. The evaluation indicators *HitRate*, *PAI* and *DCR* are used to evaluate the concentration of hotspots, while *SSI* is used to describe the shape characteristics of hotspots.

### 2.3. Study Area and Data

The comparative framework for evaluating hotspot detection approaches should consider two scenarios, i.e., the scenarios with or without the “ground truth”. The scenario with “ground truth” often corresponds to the simulation analysis. In this situation, it is easy to judge whether a cell of hotspot region is identified correctly. Both synthetic and real datasets are used to evaluate the performance of different methods quantitatively. For synthetic data, hotspot clusters of varying sizes and shapes are designed and used as the benchmarks for evaluation and comparative analysis, while the real crime data were burglaries in Philadelphia.

(1) Synthetic data

Due to a lack of knowledge about the ground truth of a hotspot, synthetic data with known hotspots need to be generated to test the effectiveness of hotspot detection methods. In existing studies, the strategies for hotspot simulation can be roughly divided into two categories: simulation based on real-world administrative boundaries [29,58,59] and based on virtual units of analysis [34,60]. The first strategy relies on the background population data and is often used to model disease clusters. The second strategy requires no additional data and focuses more on concentration characteristics of cases (e.g., the disease, crime and traffic accidents). This study adopts the second strategy and the hotspot is designed by strengthening differences between a hotspots area and a non-hotspots area. The specific implementation is described as follows.

**Figure 2:**
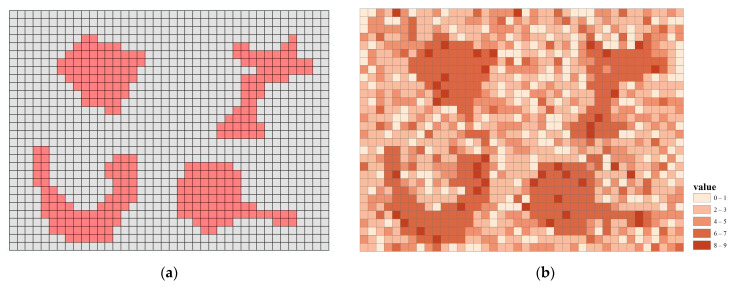
Designed hotspot area (**a**) and the final simulation data (**b**).

First, we generated a raster with 30 × 40 cells as the simulated study area. To generate clusters of hotspots, two issues should be considered. Firstly, clusters should be varying in shape. Therefore, four clusters with overly different shapes were deliberately designed, including (a) a cluster that is relatively regular and close to a circle; (b) a cluster that is elongated and irregular; (c) a cluster that is a concave polygon; and (d) a cluster that is composed of a compact shape and a slender tail. As shown in Figure 2a, the simulated hotspots comprised 279 pixels. Secondly, by definition, the hotspots should be clusters of high values. Each cell was attached with an attribute value standing for the number of crime cases that fall in the cell. To ensure the clusters of high values and conform to the characteristics of count data, the Poisson distribution was applied to generate the simulation data of crime cases (i.e., the number of crime cases). In this study, the attribute values of cells were extracted from a Poisson distribution with a mean equivalent to 3. The values of the hotspot cluster were composed of the top 5% of the upper tail of the Poisson distribution, while the values of the remaining cells were randomly taken from the same distribution (see Figure 2b).

The problem of accuracy is obviously an important one, but it is difficult to evaluate in practice. Addressing accuracy is exactly why synthetic data are needed. For synthetic data, known hotspots can be used as a benchmark for judging accuracy. In this situation, it is easy to judge whether a cell of a hotspot region is identified correctly, while a confusion matrix can be defined. The definitions of *Precision (P)*, *Recall (R)*, and *F-Measure (*$F_{1}$*)* are as follows. Then, the performance of hotspot detection can be evaluated by the following indicators:

(11) $$P=\frac{TP}{TP+FP}$$

(12) $$R=\frac{TP}{TP+FN}$$

(13) $$F_{1}=\frac{2PR}{P+R}$$

where *TP* is the number of correctly identified cells of true hotspots, *FP* is the number of cells that are not hotspots but are recognised as hotspots, and *FN* is the number of unrecognised hotspot cells. *Precision* measures the proportion of the correctly identified cells in the true hotspot; *Recall* describes the ratio of the correctly identified hotspot cells to the total cells of hotspots. *F-Measure* is the weighted harmonic average of *Precision* and *Recall* because *Precision* and *Recall* are usually inconsistent with each other. Usually, a larger $F_{1}$ stands for a better performance. For the most ideal situation, the identified hotspots are completely consistent with the ground truth, in which the recall and precision are 100%, while $F_{1}$ receives the maximum value of one.

(2) Real crime data

**Figure 3:**
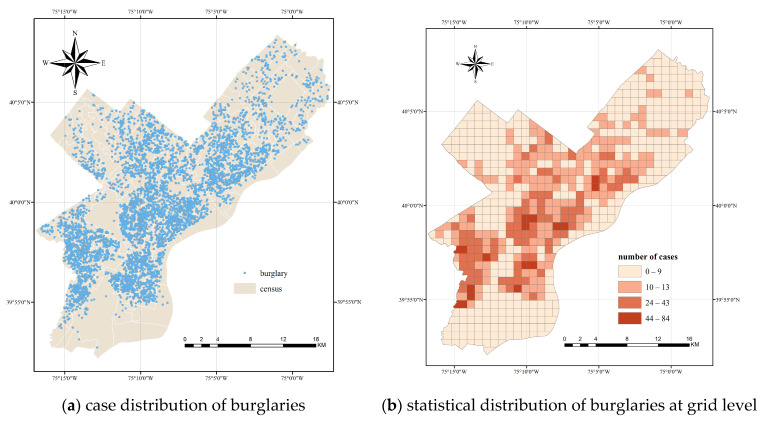
Study area and the distribution of burglaries in Philadelphia.

The performance of different methods was also evaluated by the real crime data. In this study, burglaries in Philadelphia were used as the real crime data. Philadelphia is a city in the state of Pennsylvania, USA, with an area of nearly 627.04 square kilometers. It is the sixth largest city in the United States and the most populous city in Pennsylvania. In terms of crime, Philadelphia consistently ranks above the national average. For burglary, the number of crimes reported per 100,000 persons reached 621.8. As a case study, the burglary case distribution in 2016 and the census zoning are shown in Figure 3a. All data come from the website https://www.opendataphilly.org/ (accessed on 1 May 2021).

To facilitate the visual comparison and quantitative analysis, the study area is divided into regular grids with the size of one kilometre. The grids at the boundary with areas of less than 0.5 square kilometres were merged with neighboring units, and 622 spatial units were obtained. Then, the number of cases falling in each cell were treated as the attribute value of cells. The distribution of crime cases at the grid level is shown in Figure 3b.

## 3. Results

### 3.1. Experimental Results of Synthetic Data

**Figure 4:**
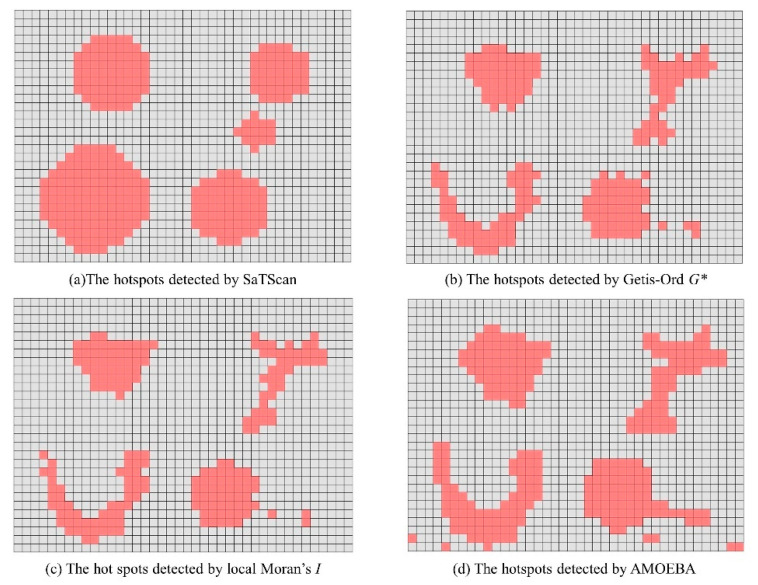
The hotspot detection result of synthetic data.

The four selected methods were applied to detect hotspots in the simulated data and the results are shown in Figure 4. The spatial neighborhood relationship is defined as queen’s case, and the upper limit of the scanning window is to cover 50% of the background population. The red cells in the figure represent the clusters with high values, i.e., the hotspots. All identified hotspots have passed the significance test at the level of *p* = 0.05.

The performance of different methods can be compared in a qualitative and quantitative way. The qualitative analysis can be achieved by visual comparison of the shape of detected hotspots. From Figure 3, it can be learned that: (a) for the SaTScan method, the detected results were heavily affected by the scanning window. For example, the circular scanning window was used to detect hotspots in this study; the shape of detected hotspots was also circular. In practice, it is difficult to determine an appropriate scanning window that may fit the real hotspots. As a result, for hotspots with complex shapes (e.g., concave polygon), detected results may have a large deviation from the real hotspots; (b) the hotspots obtained by the AMOEBA method were quite close to the pre-defined hotspots, which indicates the excellent ability to detect irregularly shaped hotspots; (c) the performance of local Moran’s *I* and Getis–Ord G* is quite similar. The methods can detect the general outline of the designed hotspots, although the results are less accurate than the AMOEBA method.

The quantitative comparison can be conducted from two perspectives. Firstly, it can be compared by the cell-based criteria, i.e., based on the number of cells that are correctly identified compared with the pre-designed hotspots. The comparison results are shown in Table 1.

The sum of *TP* and *FP* is the number of hotspot cells detected. The sum of *FN* and *FP* is the number of all misclassified cells. From Table 1, the utility of different methods can be quantitatively compared. Getis–Ord G* and Moran’s *I* methods are stricter in judging hotspots. These methods have a high precision at the cost of missing some cells. As a result, the recall of these two methods is relatively low. By contrast, the SaTScan method can achieve a higher recall, but at the expense of misjudging some cells, leading to a low precision. The SaTScan method with circular scanning windows has difficulty in dealing with hotspots with complex shapes. In general, the AMOEBA method performs better than other methods. It can achieve high precision and recall simultaneously. The overall performance of different methods can be judged by the $F_{1}$ indicator, which is the weighted harmonic average of *Precision* and *Recall*. As shown in the table, the maximum $F_{1}$ was obtained by the AMOEBA method and the minimum $F_{1}$ was obtained by the SaTScan method.

Then, the performance of various methods was further compared by the case-based criteria, i.e., based on the number of cases covered by the identified hotspots. In this study, the total number of simulated cases was 4585, which were scattered in 1200 cells. For each method, the number of captured crime cases was the sum of cases in the identified hotspot region. Then, indicators described in Section 3 could be calculated. Specifically, the indicators of the hit rate, area ratio, *PAI* and *DCR* were calculated. The final results are shown in Table 2.

In Table 2, a single indicator can hardly evaluate the performance of the method comprehensively. For example, the hotspots obtained by the SaTScan method occupied the largest area and the final hit rate reached 40.5%. However, the *PAI* of the SaTScan method was the lowest, which reflects that many non-hotspot cells are incorrectly judged as hotspot areas. Compared with the SaTScan method, the AMOEBA method could have a higher hit rate with less area, which meant that the hotspots identified by AMOEBA were “hotter” than those identified by the SaTScan method. Traditional indicators, e.g., the hit rate and *PAI* may be contradictory with each other, which made it difficult to evaluate the performance of different methods. For example, the hotspots obtained by the Getis–Ord G* and local Moran’s *I* have a higher *PAI* and lower hit rate simultaneously. This shows that methods such as Getis–Ord G* and local Moran’s *I* achieved overall high *PAI* by discarding some areas with relatively low density. We believe that the proposed *DCR* indicator is a comprehensive consideration of the hit rate and *PAI* and can reflect the concentration of hotspots. In this study, the AMOEBA method had the highest hit rate and relatively high *PAI* at the same time, therefore obtaining the highest *DCR* value. For the SaTScan method, it had a relatively high hit rate and low *PAI*, leading to the lowest density contrast ratio. It can be seen that *DCR* is a reconciled value of hit rate and *PAI*, which takes both the number of cases captured by hotspots and the “hotness” of the hotspot area into account. The higher the *DCR*, the better is the utility of the hotspot.

### 3.2. Experimental Results of Real Crime Data

**Figure 5:**
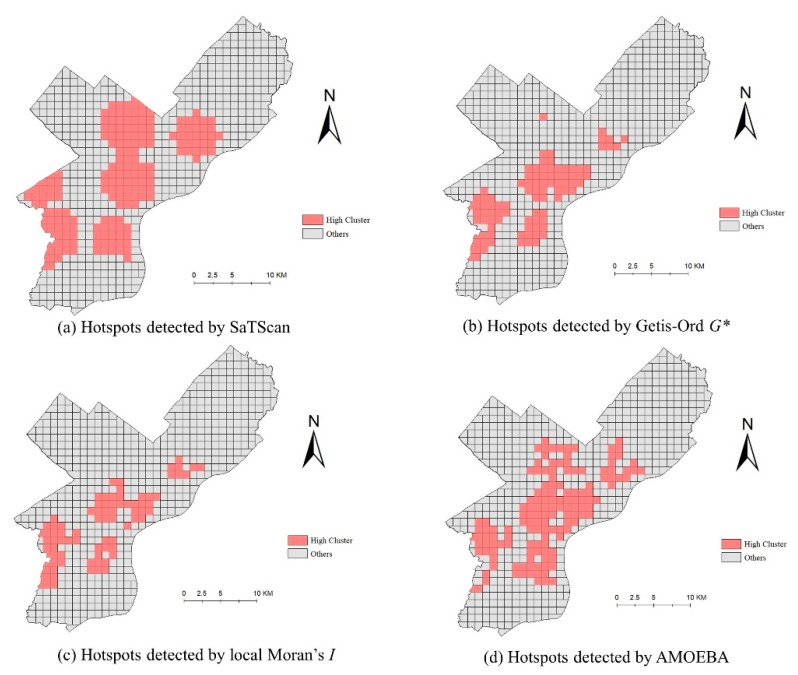
The hotspot detection result of real crime data.

Unlike with simulated data, the distribution of hotspots in real crime data is difficult to determine, in which the accuracy indicators (such as *Precision* and *Recall*) for evaluating detection results are also different. Therefore, these four types of hotspot detection methods were evaluated by comparing the concentration and shape characteristics. The significance of the results was evaluated by the significance test at the level of *p* = 0.05. The results of hotspot detection are shown in Figure 5.

As shown in Figure 5, these methods have a significant difference in terms of the hotspot shape detection ability. From a visual comparison, the local Moran’s *I* detected the smallest hotspot area, followed by the Getis–Ord G* method. The hotspots generated by SaTScan method occupied the largest area. The hotspots’ shape identified by the Getis–Ord G* method and the SaTScan method were relatively regular. Compared with the SaTScan method, hotspots identified by the AMOEBA method looked complicated. The detected hotspots had irregular shapes and were scattered in most regions of the study area. There were even some voids inside the detected hotspots. Then, the performance of these methods was quantitatively compared by calculating the adopted concentration and shape description indicators, including the hit rate, *PAI*, *DCR* and *SSI*. The results are shown in Table 3.

In Table 3, the indicators Area ratio and hit rate describe the size and general distribution of crime hotspots. From the Table, it can be learned that the size of hotspots detected by SaTScan and AMOEBA method were larger than those detected by the local Moran’s *I* and Getis–Ord G* methods. Larger hotspots corresponded to a higher hit rate. For example, the SaTScan and AMOEBA method had high hit rates and the SaTScan method received the highest hit rate (59.16%) and area ratio (28.29%) in this study. The indicators *PAI* and *DCR* described the concentration characteristic of crime hotspots. Usually, the *PAI* was negatively correlated with the size of the hotspots. For example, while the hotspots detected by local Moran’s *I* and Getis–Ord G* method had high *PAI* (3.14 and 2.87 separately), their area ratios were only 12% (the minimum) and 14%. We believe that the proposed *DCR* can describe the concentration characteristic of hotspots in a reasonable way because the *DCR* can achieve better consistency with the size characteristic of the hotspots. For example, the hotspots detected by the AMOEBA method occupied 22% of the study area, while the results obtained the highest *DCR* value. The values of the indicators were higher than the local Moran’s I and Getis–Ord G* methods. Compared with the local Moran’s *I* and Getis–Ord G* methods, the hotspots detected by the AMOEBA method captured more crime cases while maintaining a higher distribution density. The last *SSI* indicator described the shape characteristic of crime hotspots. The higher *SSI* value indicates a higher ability for describing the shape complexity of hotspots. As shown in Table 3, the hotspots obtained by the AMOEBA method had the highest *SSI* value, which means that hotspots discovered by the AMOEBA method were less restricted by the complex shape characteristic. In this sense, the performance of the AMOEBA method was superior to other selected methods in terms of ability to describe the concentration and complex shape characteristic.

## 4. Discussion

In this section, simulated and real crime datasets were used to evaluate the performance of different hotspot detection methods. For the simulated data, significant high-value clusters (i.e., the hotspots) were deliberately designed. Based on the proposed evaluation framework, the concentration and shape characteristics were used as the main comparison criteria. The result on simulated and real crime datasets exhibited good consistency. The results manifested that the AMOEBA method was more accurate in detecting complex hotspots, compared with the other three methods (i.e., the SaTScan, Local Moran’s *I* and Getis–Ord G*). The hotspots discovered by the AMOEBA method captured more cases and maintained a relatively high *PAI*, which made the AMOEBA method obtain the highest *DCR*. When crime cases were irregularly scattered throughout the research area, the AMOEBA method generated complex crime hotspots. Influenced by the scanning window, the hotspots recognized by the SaTScan method were more regular in shape. In terms of the concentration and shape compactness, the performance of the AMOEBA method and the SaTScan method was just similar to two extremes, while the local Moran’s *I* and the Getis–Ord G* methods lay between. The local Moran’s *I* and Getis–Ord G* methods performed very similarly in all respects. When these two methods identified hotspots, they not only cared about the value of the cell itself, but also the value of its neighborhood. From the experimental results on simulated data, such a strategy made these two methods tend to discard cells at the edges of hotspot clusters.

Generally, a representative hotspot detection method should have a relatively good hit rate, *DCR* and *SSI*. However, the indicators reflecting the size, concentration and shape characteristics (e.g., the hit rate, contrast density ratio and standardized shape index) are contradictory to each other to a certain extent. Overall, larger hotspot areas tend to result in higher hit rates and lower *PAI*. *DCR* reconciles the contradiction between the two to a certain extent and becomes a comprehensive consideration of hotspot concentration. At the same time, higher *DCR* may imply more complex shape features. Because a relatively high *DCR* needs to occupy a small area while maintaining a relatively high hit rate, this means that less concentrated neighborhood units need to be discarded, resulting in complex shape features. The selected hotspot detection method has its own advantages and disadvantages when dealing with these requirements. Although the AMOEBA method was more powerful in discovering complex hotspots, it is difficult to conclude which method is always optimal. The selection of hotspot detection methods should consider the distribution characteristics of the data and application scenarios. For example, the detected hotspots may have a great influence on police practice and resource deployment. When police resources are limited, overly complicated hotspots would cause inconveniences to police practice. The identification of hotspots and deployment of police resources should be jointly considered. In this aspect, when selecting a suitable hotspot detection method, our suggestion is as follows. If the distribution of crime cases is relatively concentrated and there are obvious clusters, the AMOEBA method will be a good choice. If the distribution of crime cases is irregularly scattered, the SaTScan method and the Getis–Ord G* method may be a suitable choice because these two methods would generate regular-shaped hotspots, which may provide more convenience for police practice and resource deployment. However, if the aim of hotspot detection is to explore the causes and influencing factors of crime hotspots, it is necessary to know the accurate distribution of hotspots. In this situation, the AMOEBA method is more appropriate.

## 5. Conclusions

In this study, a comparative study of spatial hotspot detection methods was conducted by considering the concentration and shape characteristics. Firstly, we established an evaluation framework for crime hotspot detection, which can be applied to situations either with or without evaluation benchmarks. Secondly, two additional evaluation indicators were defined considering the concentration and morphological characteristics of hotspots. These defined indicators can be used as a supplement to the existing evaluation indicators. Finally, four classic spatial hotspot detection methods were quantitatively compared using synthetic and real crime data. Results show that the proposed evaluation framework and indicators can describe the size, concentration and shape characteristics of detected hotspots, thus supporting the quantitative comparison of different methods. Among the four methods, the AMOEBA method is more accurate in describing the concentration and shape characteristics and is more powerful in discovering complex hotspots.

Although the evaluation framework is mainly designed for spatial hotspots, the general framework may be extended to evaluate spatio–temporal hotspot detection methods by supplementing indicators for spatio–temporal hotspots. Essentially, detection of spatial hotspots can serve as a fundamental step for spatio–temporal hotspots. It should be also noted that one aim of crime hotspot detection is to support crime prediction and crime intervention. Despite the fact that hotspot detection is mainly based on retrospective crime data, crime hotpot detection often serves as an effective tool for crime prediction because crime hotspots are often stable for a period of time. The reason behind that is that the existence of crime hotspots is often related to a collection of influential factors (e.g., the social–economic condition, building environment and human mobility), and these factors will not change drastically in the short term [61,62,63]. In this study, we mainly focused on the performance of various methods in crime spatial hotspot detection; the research can be extended to crime prediction by considering both retrospective and future occurrence of crime.

In future research, some issues require further examination. Firstly, more datasets and hotspot detection methods should be compared to prove the feasibility of the evaluation framework, especially with the spatio–temporal hotspot detection methods. Secondly, the criteria for judging the performance of hotspot detection methods may not be just the size, concentration and shape characteristics. More aspects should be jointly considered to evaluate the performance of hotspot detection methods comprehensively. In addition, the relationship between the precision of hotspot detection and application should be further considered.

## Figures and Tables

**Table 1:** Cell-based evaluation results of hotspots on synthetic data.

Methods	*TP + FP*	*FN + FP*	*FN*	*FP*	*TP*	*Precision*	*Recall*	$F_{1}$
SaTScan	320	137	48	89	231	72.1%	82.8%	0.771
Getis–Ord G*	209	80	75	5	204	97.6%	73.1%	0.836
local Moran’s *I*	198	81	81	0	198	100%	71%	0.830
AMOEBA	286	7	0	7	279	97.6%	100%	0.988

**Table 2:** Case-based evaluation results of hotspots on synthetic data.

Methods	*n*	*HitRate*	*AreaRatio*	*PAI*	*DCR*
SaTScan	1857	40.5%	26.67%	1.52	1.87
Getis–Ord G*	1393	30.4%	17.42%	1.75	2.07
local Moran’s *I*	1352	29.5%	16.50%	1.78	2.12
AMOEBA	1906	41.6%	23.83%	1.74	2.27

**Table 3:** Evaluation results of hotspots on real crime data.

Methods	*n*	*HitRate*	*AreaRatio*	*PAI*	*DCR*	*SSI*
SaTScan	4139	59.16%	28.29%	2.06	3.67	0.678
Getis–Ord G*	2953	42.21%	14.65%	2.87	4.26	0.698
local Moran’s *I*	2748	39.29%	12.65%	3.14	4.67	0.742
AMOEBA	4120	58.89%	22.33%	2.60	4.99	0.787

## Data Availability

Publicly available datasets were analyzed in this study. This data can be found here: https://www.opendataphilly.org/ (accessed on 1 May 2022).
